# Exploration of the application potential of serum multi-biomarker model in colorectal cancer screening

**DOI:** 10.1038/s41598-024-60867-0

**Published:** 2024-05-02

**Authors:** Runhao Xu, Jianan Shen, Yan Song, Jingbo Lu, Yijing Liu, Yun Cao, Zhenhua Wang, Jie Zhang

**Affiliations:** 1 Department of Clinical Laboratory, Renji Hospital Affiliated to Shanghai Jiao Tong University School of Medicine, Shanghai, 200001 China; 2 Department of Gastroenterology, Renji Hospital Affiliated to Shanghai Jiao Tong University School of Medicine, Shanghai, 200001 China; 3 Present Address: 145 Middle Shandong Road, Shanghai, China

**Keywords:** Colorectal cancer, Lipid, Bile acid, Screening model, Lipids, Diagnostic markers, Cancer screening

## Abstract

Analyzing blood lipid and bile acid profile changes in colorectal cancer (CRC) patients. Evaluating the integrated model's diagnostic significance for CRC. Ninety-one individuals with colorectal cancer (CRC group) and 120 healthy volunteers (HC group) were selected for comparison. Serum levels of total cholesterol (TC), triglycerides (TG), high-density lipoprotein cholesterol (HDL-C), low-density lipoprotein cholesterol (LDL-C), and apolipoproteins (Apo) A1, ApoA2, ApoB, ApoC2, and ApoC3 were measured using immunoturbidimetric and colorimetric methods. Additionally, LC–MS/MS was employed to detect fifteen bile acids in the serum, along with six tumor markers: carcinoembryonic antigen (CEA), carbohydrate antigens (CA) 125, CA19-9, CA242, CA50, and CA72-4. Group comparisons utilized independent sample *t*-tests and Mann–Whitney *U* tests. A binary logistic regression algorithm was applied to fit the indicators and establish a screening model; the diagnostic accuracy of individual Indicators and the model was analyzed using receiver operating characteristic (ROC) curves. The CRC group showed significantly lower levels in eight serum lipid indicators and eleven bile acids compared to the HC group (P < 0.05). Conversely, serum levels of TG, CA19-9, and CEA were elevated (P < 0.05). Among the measured parameters, ApoA2 stands out for its strong correlation with the presence of CRC, showcasing exceptional screening efficacy with an area under the curve (AUC) of 0.957, a sensitivity of 85.71%, and a specificity of 93.33%. The screening model, integrating ApoA1, ApoA2, lithocholic acid (LCA), and CEA, attained an impressive AUC of 0.995, surpassing the diagnostic accuracy of individual lipids, bile acids, and tumor markers. CRC patients manifest noteworthy alterations in both blood lipids and bile acid profiles. A screening model incorporating ApoA1, ApoA2, LCA, and CEA provides valuable insights for detecting CRC.

## Introduction

According to statistics from the International Agency for Research on Cancer (IARC) of the World Health Organization, there were 1.93 million new cases of colorectal cancer (CRC) worldwide in 2020, with approximately 930,000 deaths. CRC ranks third in incidence among all cancers and second in mortality^1^. In recent years, a concerning trend has emerged with a rise in CRC cases among younger age groups, where incidence rates have doubled in individuals under 50 years old^2^. Regarding survival rates, early-stage CRC has a 5-year survival rate exceeding 90%, whereas it plummets to below 10% in late-stage cases^3^. Therefore, emphasizing early screening is crucial in reducing CRC mortality rates.

Colonoscopy is widely regarded as the gold standard for both screening and diagnosing CRC. Early detection of high-risk individuals and prompt intervention play a pivotal role in lowering CRC mortality rates and positively impacting treatment outcomes^4^. Nevertheless, colonoscopy, being an invasive procedure, carries inherent risks like bleeding and perforation. The necessity for meticulous bowel preparation can contribute to an unfavorable patient experience, diminishing willingness to adhere to regular follow-ups. These factors, in turn, constrain the broad utilization of colonoscopy for early CRC screening^5,6^. Presently, entities like the American Gastroenterological Association and the Chinese Medical Association's Society of Gastroenterology advocate for a "two-step" screening approach for CRC^7,8^. The initial phase employs low-invasive screening techniques like hematological tests and multi-target stool DNA testing. Hematological tests, known for their simplicity, speed, safety, and minimally invasive, can capture evolving changes in CRC progression, proving valuable in screening and identifying high-risk individuals within ostensibly healthy populations. The subsequent stage entails a comprehensive colonoscopy, strategically optimizing colonoscopy resources and playing an important role in the early detection and intervention of CRC.

Recent studies reveal that alterations in blood lipids are a distinctive characteristic observed in various malignancies, including CRC patients^9^. Bile acids, metabolic products of cholesterol in the liver and processed in the intestine^10^, have been implicated in the risk of CRC due to disruptions in lipid and bile acid metabolism^11,12^. In this study, we assessed a panel of 9 serum lipids, encompassing total cholesterol (TC), triglycerides (TG), high-density lipoprotein cholesterol (HDL-C), low-density lipoprotein cholesterol (LDL-C), and apolipoproteins (Apo) A1, ApoA2, ApoB, ApoC2, and ApoC3, alongside 15 serum bile acids and 6 tumor markers including carcinoembryonic antigen (CEA), carbohydrate antigens (CA) 125, CA19-9, CA242, CA50, and CA72-4. This investigation involved 91 CRC patients and 120 healthy volunteers. The application of a data analysis model was employed to explore its value in identifying CRC.

## Materials and methods

### Study subjects and enrollment criteria

Samples were collected from August 2022 to February 2023 at Renji Hospital affiliated with Shanghai Jiao Tong University School of Medicine. The study participants included 91 CRC patients, comprising the CRC group, and 120 healthy volunteers, forming the healthy control (HC) group. Inclusion criteria for the CRC group were as follows: patients newly diagnosed with primary CRC through histopathological examination, who had not undergone radiation, chemotherapy, or surgical treatment. All of the CRC patients were adenocarcinoma. Healthy volunteers were recruited from individuals undergoing medical check-ups at our center. After excluding colorectal cancer and other gastrointestinal diseases through colonoscopy, as well as excluding malignant tumors, inflammation, and cardiovascular diseases through thoracic CT scans, electrocardiograms, abdominal ultrasounds, and a comprehensive evaluation including fecal occult blood tests and blood examinations (such as routine blood tests, liver function, renal function, etc.), they were defined as healthy. From October 2023 to December 2023, we collected an additional 22 CRC patients and 22 healthy volunteers, matched based on the same inclusion criteria, to form an external validation set. There were no statistically significant differences in age and gender between the groups (*P* > 0.05).

### Methods

#### Sample preprocessing

Four milliliters of fasting whole blood samples were collected using red top serum separator tube (Gongdong, China). These samples were centrifuged at 2685 × g for 10 min to separate the serum, with lipemic samples excluded. The serum was then stored at − 80 °C until analysis to avoid repeated freeze–thaw cycles. We utilized approximately one milliliter of serum for testing various indicators, strictly following the manufacturer's instructions throughout the testing process.

#### Lipid indicator measurement

Levels of TC, TG, HDL-C, LDL-C, ApoA1, ApoA2, ApoB, ApoC2, and ApoC3 were measured using the H7600 Series Automatic Analyzer (Hitachi, Japan). Reagent kits for ApoA1 and ApoB, utilizing immunoturbidimetric methods, were obtained from Maccura Biotechnology Co., Ltd. Similarly, reagent kits for ApoA2, ApoC2, and ApoC3, utilizing immunoturbidimetric methods, were obtained from Beijing Leadman Biochemistry Co., Ltd. Reagent kits for TC, TG, HDL-C, and LDL-C, utilizing colorimetric methods, were obtained from Fujifilm Wako Pure Chemical Co., Ltd., Japan.

#### Detection of bile acid profile

The bile acid profile was detected using liquid chromatography-tandem mass spectrometry (LC–MS/MS) with the API3200MD triple quadrupole mass spectrometer (ABSciex, USA) and Shimadzu series liquid chromatograph (Shimadzu, Japan). The reagent kits were purchased from Shanghai ClinMeta Co., Ltd. The assay comprised 15 components: cholic acid (CA), glycocholic acid (GCA), taurocholic acid (TCA), chenodeoxycholic acid (CDCA), glycochenodeoxycholic acid (GCDCA), taurochenodeoxycholic acid (TCDCA), deoxycholic acid (DCA), glycodeoxycholic acid (GDCA), taurodeoxycholic acid (TDCA), lithocholic acid (LCA), glycolithocholic acid (GLCA), taurolithocholic acid (TLCA), ursodeoxycholic acid (UDCA), glycoursodeoxycholic acid (GUDCA), and tauroursodeoxycholic acid (TUDCA).

#### Detection of tumor markers

CEA, CA125, CA19-9, and CA72-4 were assayed using the Cobas e801 automatic analyzer (Roche, Switzerland), with corresponding reagent kits. CA50 and CA242 were analyzed using the Maglumi2000 automatic analyzer (Snibe, China), also with corresponding reagent kits. Chemiluminescence was the method of detection for all markers.

### Statistical methods

Statistical analyses were performed using SPSS 17.0 and GraphPad 7.0. The Kolmogorov–Smirnov test was utilized for normality testing. Quantitative data with a normal distribution were reported as mean ± standard deviation ($\overline{{\text{x}} }$ ± s) and compared using independent sample *t*-tests. Non-normally distributed quantitative data were presented as median (Q1, Q3) and analyzed using Mann–Whitney *U* test. A *P*-value less than 0.05 was considered statistically significant.

**Figure 1:**
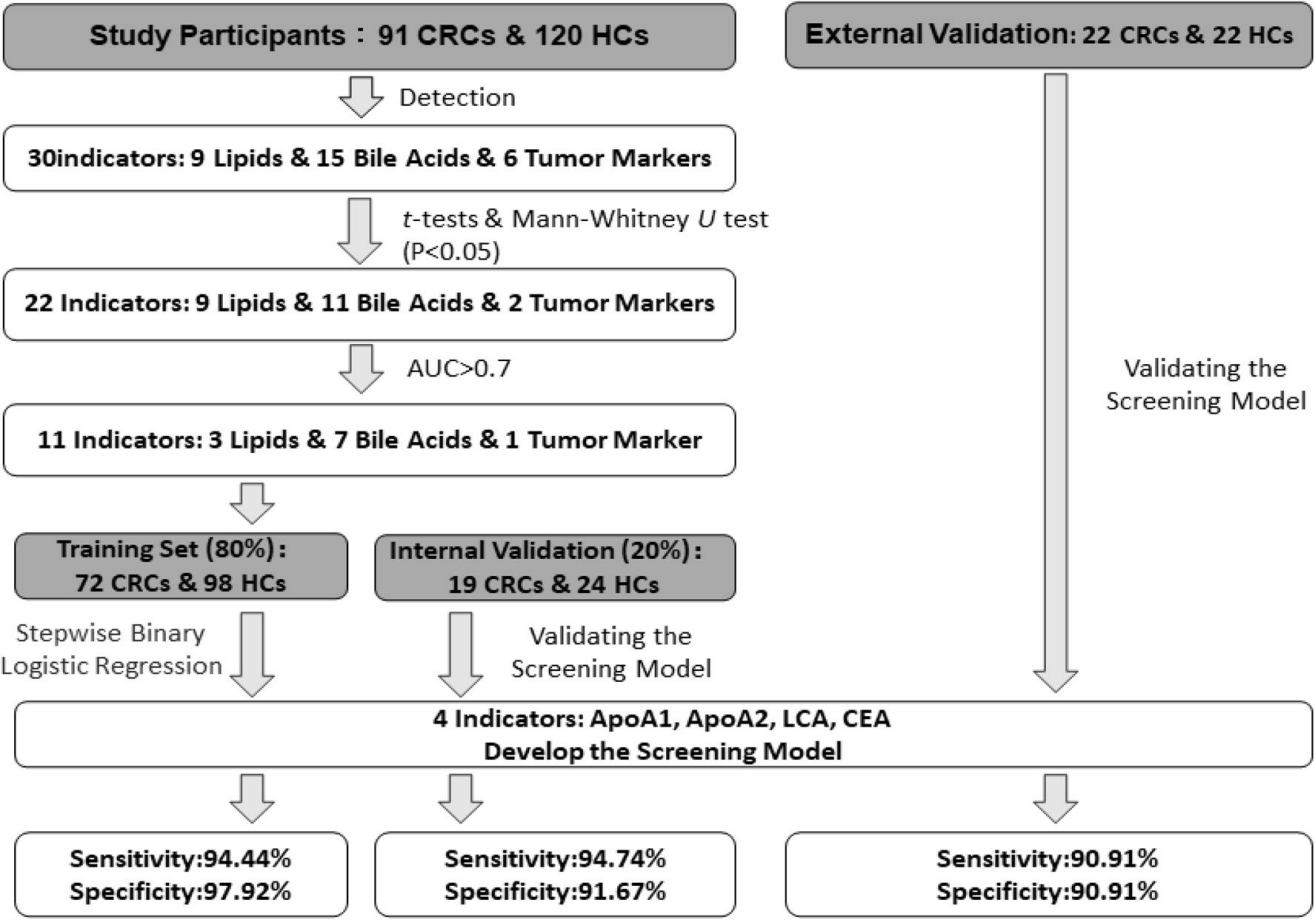
Flow diagram for development of the CRC screening model. *HC* Healthy control, *CRC* Colorectal cancer.

The indicators showing statistically significant differences between CRC group and HC group with AUC greater than 0.7 would be selected for developing a stepwise binary logistic regression model (Backward Likelihood Ratio method). We randomly divided participants' samples (by receiving date) such that 80% formed the training set and 20% formed the internal validation set. At each step, the indicator with the least contribution to the model's likelihood was removed. The process continues until the likelihood ratio test indicated that removing any further indicators would significantly change the model’s fit (*P*-value < 0.05). The remaining indicators were considered the best subset, which composed the optimal model. The model's cut-off value was established at 0.5, where values exceeding 0.5 were categorized as CRC, while values below 0.5 were categorized as HC. Omnibus test was used to assesses the overall significance of the model. When the *P*-value of the Omnibus Test was less than 0.05, it indicated that at least one indicator was significant. Hosmer–Lemeshow test was used to assess the goodness-of-fit of the model. When the *P*-value of the Hosmer–Lemeshow test was greater than 0.05, it indicated model's classification forecasting were consistent with reality. The diagnostic accuracy of individual indicators and the model was assessed using receiver operating characteristic (ROC) curves. Flow diagram for development of the CRC screening model had been shown in Fig. 1.

### Ethics declarations

This study received approval from the Ethics Committee of Renji Hospital Affiliated with Shanghai Jiao Tong University School of Medicine (case number RA-2022-335) and adhered to the principles outlined in the Declaration of Helsinki. Prior to their inclusion in the study, all patients provided informed consent.

## Results

### Characteristics of the study populations

The median age of participants in the HC and CRC groups was 65 and 66 years old, respectively. Males comprised 64.17% and 63.73% of the HC and CRC groups, respectively. Early and middle-stage tumors (Stages I, II, and III) were prevalent in the CRC group, accounting for 87.91%. Late-stage tumors (Stage IV) represented 12.09% of the CRC cases. Additionally, the tumor location distribution revealed 42.86% in the rectum and 57.14% in the colon. As the Table 1 showed.

**Table 1:** Clinical characteristics of the participants.

Group	Study participants		External validation	
	HC	CRC	HC	CRC
Total (%)	120 (100.0)	91 (100.0)	22 (100.0)	22 (100.0)
Age distribution				
< 50 (%)	9 (7.5)	6 (6.6)	4 (18.2)	3 (13.6)
50–59 (%)	38 (31. 7)	16 (17.6)	8 (36.3)	9 (40.9)
60–69 (%)	42 (35.0)	38 (41.8)	6 (27.3)	4 (18.2)
> 70 (%)	31 (25.8)	31 (34.0)	4 (18.2)	6 (27.3)
Median (years)	65.00	66.00	58.00	59.00
Mean (years)	63.37	65.04	59.09	61.18
SD	8.99	9.86	10.65	11.70
Gender distribution				
Male (%)	77 (64.2)	58 (63.7)	14 (63.6)	14 (63.6)
Female (%)	43 (35.8)	33 (36.3)	8 (36.4)	8 (36.4)
Stage distribution^a^				
I (%)	–	23 (25.3)	–	7 (31.8)
II (%)	–	25 (27.5)	–	8 (36.4)
III (%)	–	32 (35.1)	–	6 (27.3)
IV (%)	–	11 (12.1)	–	1 (4.5)
Tumor location distribution				
Rectum (%)	–	39 (42.9)	–	8 (36.4)
Colon (%)	–	52 (57.1)	–	14 (63.6)

*HC* Healthy control, *CRC* Colorectal cancer.

^a^According to American Joint Committee on Cancer (AJCC) Cancer Staging Manual Eighth Edition.

### Comparison of 9 lipid indicators between HC and CRC groups

**Figure 2:**
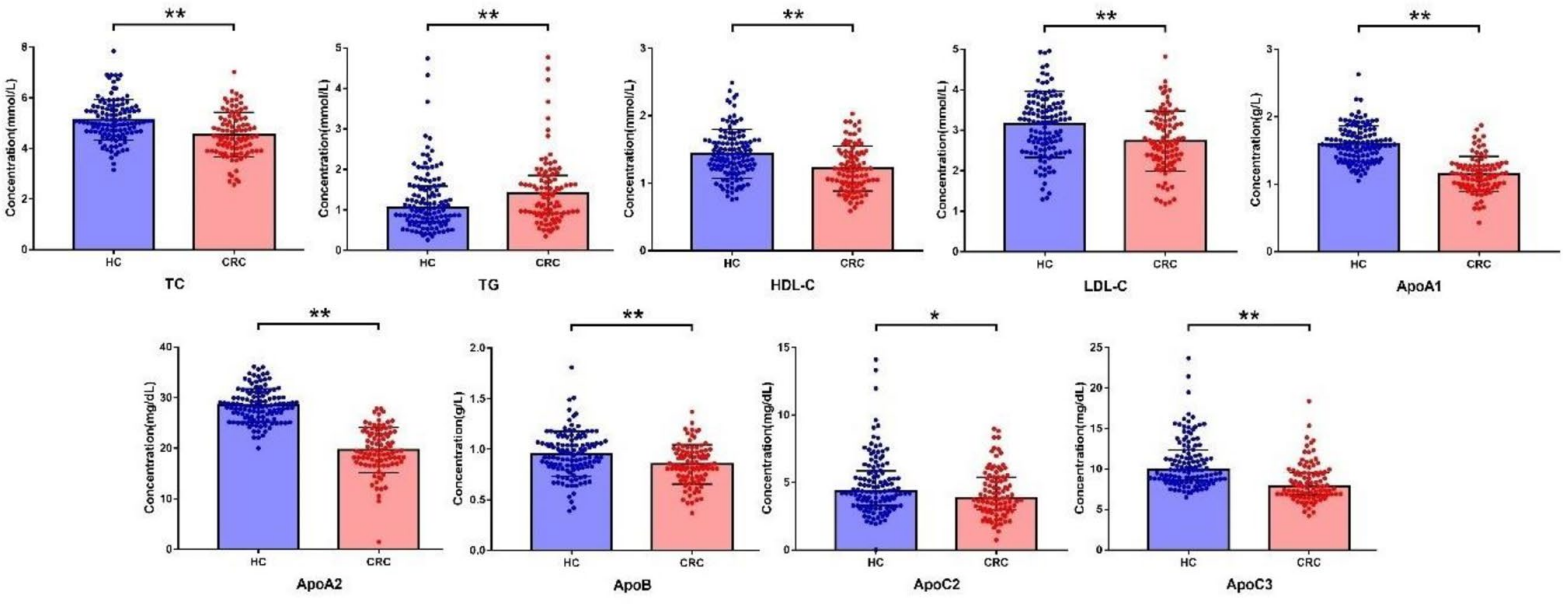
Comparison of lipid Indicators levels between two groups. *HC* Healthy control, *CRC* Colorectal cancer; *P < 0.05; **P < 0.01; Error bars (TC, HDL-C, LDL-C, ApoA1, ApoA2, ApoB): Mean with standard deviation; Error bars (TG, ApoC2, ApoC3): Median with interquartile range.

In the CRC group, the serum levels of TC, HDL-C, LDL-C, ApoA1, ApoA2, ApoB, ApoC2 and ApoC3 were significantly lower than those in the HC group (*P* < 0.05). The level of TG was higher in the CRC group compared to the HC group (*P* < 0.05), as shown in Table 2 and Fig. 2.

**Table2:** Comparison of Indicators levels between two groups.

Indicators	Group HC (120 samples)	Group CRC (91 samples)	*P* value
TC (mmol/L)	5.13 ± 0.81	4.54 ± 0.89	< 0.001
TG (mmol/L)	1.05 (0.70 ~ 1.59)	1.40 (0.92 ~ 1.85)	0.009
HDL-C (mmol/L)	1.43 ± 0.36	1.22 ± 0.33	< 0.001
LDL-C (mmol/L)	3.14 ± 0.81	2.73 ± 0.75	< 0.001
ApoA1 (g/L)	1.60 ± 0.27	1.15 ± 0.26	< 0.001
ApoA2 (mg/dL)	28.49 ± 3.31	19.64 ± 4.48	< 0.001
ApoB (g/L)	0.95 ± 0.22	0.85 ± 0.19	< 0.001
ApoC2 (mg/dL)	4.36 (3.29 ~ 5.87)	3.86 (2.98 ~ 5.40)	0.039
ApoC3 (mg/dL)	9.87 (8.61 ~ 12.35)	7.87 (6.82 ~ 9.58)	< 0.001
CA (nmol/L)	83.07 (41.17 ~ 205.32)	41.74 (16.24 ~ 112.50)	< 0.001
GCA (nmol/L)	137.69 (72.37 ~ 310.24)	106.34 (42.74 ~ 346.16)	0.289
TCA (nmol/L)	14.35 (7.19 ~ 33.23)	12.31 (6.09 ~ 37.80)	0.579
CDCA (nmol/L)	617.46 (262.30 ~ 1086.87)	114.80 (30.06 ~ 482.35)	< 0.001
GCDCA (nmol/L)	661.50 (379.11 ~ 1216.48)	668.91 (265.29 ~ 1336.94)	0.495
TCDCA (nmol/L)	56.85 (34.79 ~ 120.48)	65.57 (27.13 ~ 154.63)	0.700
DCA (nmol/L)	409.55 (224.81 ~ 672.04)	65.64 (8.59 ~ 249.18)	< 0.001
GDCA (nmol/L)	226.13 (112.92 ~ 409.32)	70.49 (6.22 ~ 247.13)	< 0.001
TDCA (nmol/L)	31.21 (17.09 ~ 66.26)	7.23 (1.14 ~ 26.04)	< 0.001
LCA (nmol/L)	27.99 (18.13 ~ 45.13)	5.68 (0.40 ~ 13.14)	< 0.001
GLCA (nmol/L)	7.01 (3.29 ~ 17.82)	1.22 (0.01 ~ 3.92)	< 0.001
TLCA (nmol/L)	1.14 (0.51 ~ 1.94)	0.41 (0.23 ~ 1.13)	< 0.001
UDCA (nmol/L)	110.08 (46.78 ~ 198.86)	14.43 (0.24 ~ 89.30)	< 0.001
GUDCA (nmol/L)	137.02 (68.75 ~ 301.37)	48.67 (13.10 ~ 200.27)	< 0.001
TUDCA (nmol/L)	3.87 (1.72 ~ 7.81)	0.01 (0.01 ~ 4.64)	< 0.001
CA125 (U/mL)	12.50 (8.47 ~ 16.48)	11.30 (7.67 ~ 16.40)	0.420
CA19-9 (U/mL)	8.31 (5.92 ~ 11.85)	11.30 (6.07 ~ 18.20)	0.004
CA242 (U/mL)	5.18 (3.47 ~ 8.07)	5.14 (3.00 ~ 12.52)	0.458
CA50 (U/mL)	5.53 (3.48 ~ 9.07)	6.15 (3.95 ~ 11.69)	0.100
CA72-4 (U/mL)	1.61 (1.50 ~ 2.75)	1.68 (1.50 ~ 3.95)	0.206
CEA (ng/mL)	1.48 (0.95 ~ 2.22)	3.07 (1.80 ~ 7.91)	< 0.001

*HC* Healthy control, *CRC* Colorectal cancer, Compare to Group HC.

### Comparison of 15 bile acids between HC and CRC groups

**Figure 3:**
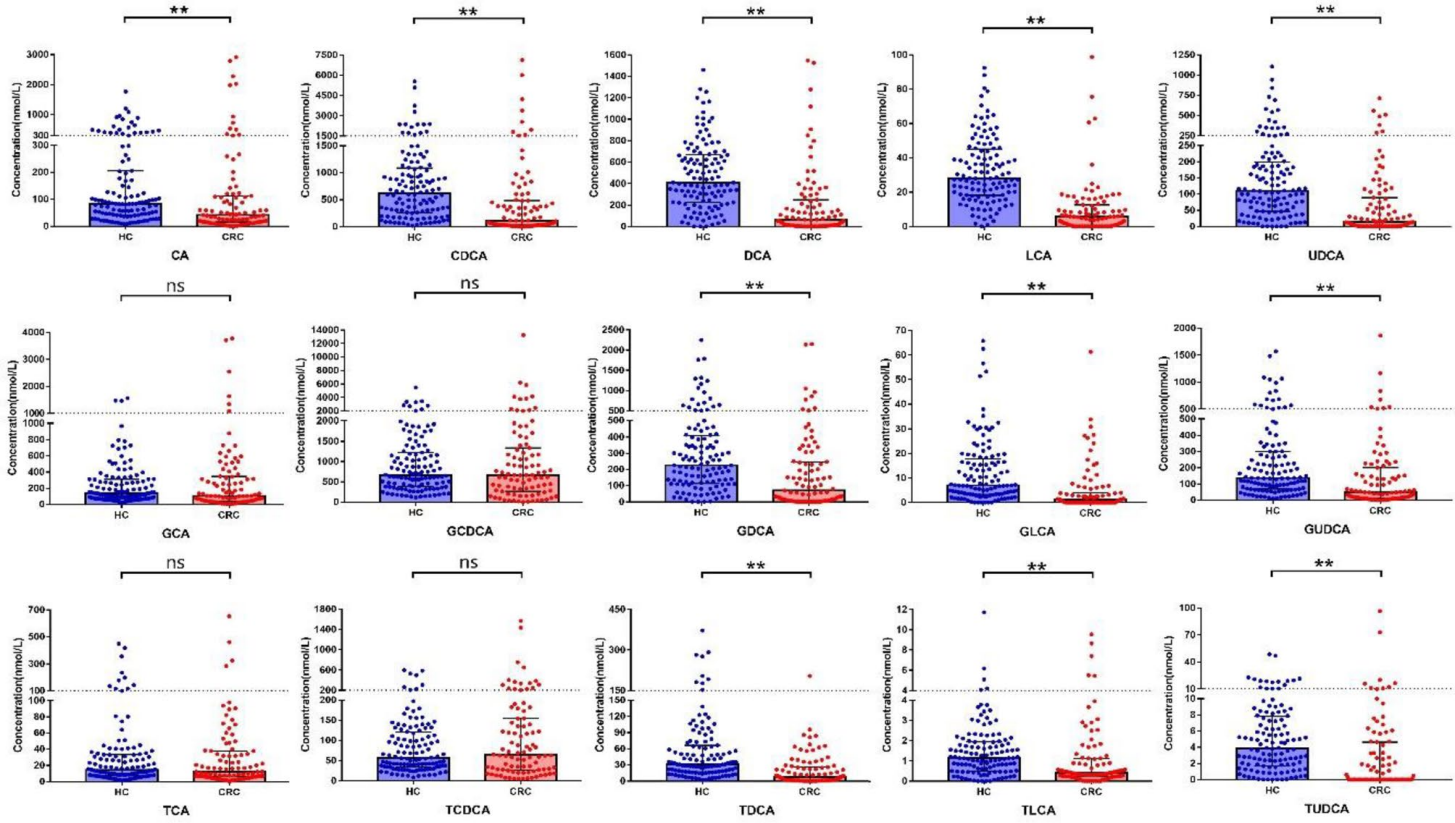
Comparison of bile acids levels between two groups. *HC* Healthy control, *CRC* Colorectal cancer; *P < 0.05; **P < 0.01; ns (no statistically significant): *P* > 0.05; Error bars: Median with interquartile range.

In the CRC group, the serum levels of free primary bile acids including CA, CDCA, as well as secondary bile acids such as DCA, GDCA, TDCA, LCA, GLCA, TLCA, UDCA, GUDCA, TUDCA, were significantly lower than those in the HC group (*P* < 0.05). However, there were no statistically significant differences in the levels of conjugated primary bile acids, including GCA, TCA, GCDCA, TCDCA between the two groups (*P* > 0.05), as shown in Table 2 and Fig. 3.

### Comparison of 6 gastrointestinal tumor markers between HC and CRC groups

**Figure 4:**
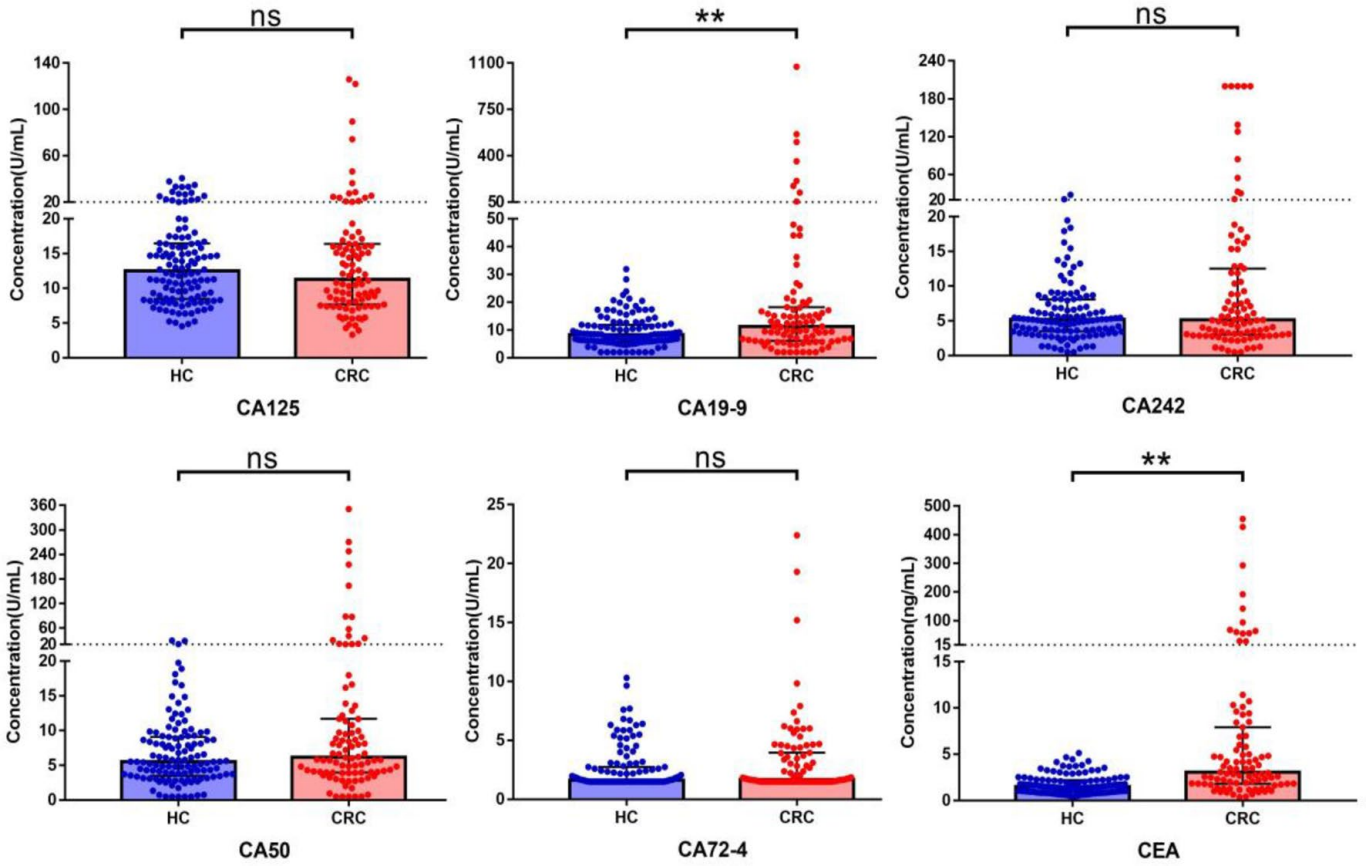
Comparison of tumor markers levels between two groups. *HC* Healthy control, *CRC* Colorectal cancer; *P < 0.05; **P < 0.01; ns (no statistically significant): *P* > 0.05; Error bars: Median with interquartile range.

In the CRC group, the serum levels of CA19-9 and CEA were significantly higher than those in the HC group (*P* < 0.05). However, no statistically significant differences were observed in the levels of CA125, CA242, CA50, and CA72-4 between the two groups (*P* > 0.05), as shown in Table 2 and Fig. 4.

### Evaluation of diagnostic accuracy for CRC

Statistically significant differences were observed between the HC and CRC groups in 22 indicators, including serum TC, TG, HDL-C, LDL-C, ApoA1, ApoA2, ApoB, ApoC2, ApoC3, CA, CDCA, DCA, GDCA, TDCA, LCA, GLCA, TLCA, UDCA, GUDCA, TUDCA, CA19-9, CEA. The diagnostic accuracy for CRC was evaluated using ROC curves. Eleven indicators, including ApoA1, ApoA2, ApoC3, CDCA, DCA, TDCA, LCA, GLCA, UDCA, TUDCA, and CEA, had an area under the curve (AUC) greater than 0.7. Among these, ApoA2 showed the best diagnostic accuracy, with an AUC of 0.957, a sensitivity of 85.71%, a specificity of 93.33%, and the largest Youden's Index of 0.79. Detailed results were presented in Table 3 and Supplementary Table 3.

**Table 3:** Performance of the indicators in screening CRC.

Indicators	AUC (95% CI)	Optimal Cut-off value	Sensitivity (%)	Specificity (%)	Youden’s Index
TC	0.688 (0.621 ~ 0.750)	4.49 mmol/L	52.75	81.67	0.344
TG	0.605 (0.536 ~ 0.672)	1.37 mmol/L	50.55	69.17	0.197
HDL-C	0.672 (0.604 ~ 0.735)	1.24 mmol/L	58.24	70.83	0.291
LDL-C	0.649 (0.580 ~ 0.713)	3.16 mmol/L	72.53	54.17	0.267
ApoA1	0.893 (0.843 ~ 0.931)	1.32 g/L	82.42	85.00	0.674
ApoA2	0.957 (0.920 ~ 0.980)	24.18 mg/dL	85.71	93.33	0.790
ApoB	0.640 (0.571 ~ 0.705)	1.02 g/L	84.62	39.17	0.238
ApoC2	0.583 (0.513 ~ 0.650)	3.86 mg/dL	50.55	66.67	0.172
ApoC3	0.748 (0.684 ~ 0.805)	7.98 mg/dL	52.75	87.50	0.403
CA	0.642 (0.574 ~ 0.707)	52.17 nmol/L	59.34	67.50	0.268
CDCA	0.732 (0.666 ~ 0.790)	118.74 nmol/L	52.75	88.33	0.411
DCA	0.796 (0.735 ~ 0.848)	166.34 nmol/L	69.23	83.33	0.526
GDCA	0.694 (0.627 ~ 0.755)	104.21 nmol/L	56.04	80.83	0.369
TDCA	0.777 (0.715 ~ 0.832)	12.38 nmol/L	60.44	85.00	0.454
LCA	0.876 (0.824 ~ 0.918)	14.38 nmol/L	86.81	76.67	0.635
GLCA	0.767 (0.704 ~ 0.822)	2.96 nmol/L	70.33	78.33	0.487
TLCA	0.678 (0.611 ~ 0.741)	0.58 nmol/L	65.93	74.17	0.401
UDCA	0.745 (0.681 ~ 0.803)	42.68 nmol/L	65.93	78.33	0.443
GUDCA	0.695 (0.628 ~ 0.756)	56.42 nmol/L	54.95	83.33	0.383
TUDCA	0.730 (0.665 ~ 0.789)	0.06 nmol/L	52.75	95.83	0.486
CA19-9	0.615 (0.546 ~ 0.681)	8.84 U/mL	63.74	60.83	0.246
CEA	0.789 (0.728 ~ 0.842)	2.52 ng/mL	62.64	83.33	0.460

*CI* Confidence interval, Optimal Cutoff Value: Cut-off value that maximizes the sum of sensitivity and specificity; Youden’s Index: Sensitivity + Specificity − 1.

### Development and validation of the CRC screening model

#### Establishment of the model

Eighty percent of study participants’ samples were selected for forming the training set according to receiving date, including 96 cases from the HC group and 72 cases from the CRC group. Clinical characteristics of them were shown in Supplementary Table 4. The 11 indicators with AUC greater than 0.7 were included in a stepwise binary logistic regression analysis (Backward Likelihood Ratio method). When ApoA1 was removed in step 9, statistically significant difference was observed between model 8 and model 9(*P* = 0.024). Thus model 8 which composed of ApoA1, ApoA2, LCA, and CEA was considered as the final screening model. The model is represented as Y = 1/(1 + ${e}^{-{\text{Logit}}({\text{P}})})$, where Logit (*P*) = − 4.847 × ApoA1 − 1.041 × ApoA2 − 0.132 × LCA + 2.233 × CEA + 30.691. Omnibus test (*χ*^2^ = 200.79, *P* = 0.00) showed that indicators in the model were significant and Hosmer–Lemeshow test (*χ*^2^ = 0.93, *P* = 0.99) showed that the model's classification forecasting are relatively consistent with reality. The diagnostic accuracy of each step of the stepwise binary logistic regression was shown in Table 4. The steps of developing the screening model were shown in Supplementary Table 5.

**Table 4:** Performance of the stepwise binary logistic regression models in screening CRC.

Step (model)	Model’s components	Removing indicator	AUC (95% CI)	Cut-off	Sensitivity (%)	Specificity (%)	Youden’s index
1	11 Indicators	DCA	0.997 (0.973 ~ 0.999)	0.5	95.83	97.92	0.938
2	10 Indicators	TUDCA	0.997 (0.973 ~ 0.999)	0.5	95.83	97.92	0.938
3	9 Indicators	TDCA	0.997 (0.973 ~ 0.999)	0.5	95.83	97.92	0.938
4	8 Indicators	UDCA	0.996 (0.971 ~ 0.999)	0.5	95.83	96.87	0.927
5	7 Indicators	CDCA	0.995 (0.970 ~ 0.999)	0.5	95.83	96.87	0.927
6	6 Indicators	APOC3	0.995 (0.969 ~ 0.999)	0.5	94.44	97.92	0.927
7	5 Indicators	GLCA	0.995 (0.969 ~ 0.999)	0.5	94.44	97.92	0.924
**8**	**4 Indicators**	**ApoA1**	**0.995 (0.969 ~ 0.999)**	**0.5**	**94.44**	**97.92**	**0.924**
9	3 Indicators	LCA	0.993 (0.966 ~ 0.999)	0.5	91.67	97.92	0.896
10	2 Indicators	CEA	0.979 (0.944 ~ 0.995)	0.5	91.67	93.75	0.854
11	1 Indicator	–	0.964 (0.924 ~ 0.987)	0.5	87.50	93.75	0.812

*CI* Confidence interval, Removing indicator: The indicator would be removed in next step. Cut-off: Probability of occurrence; Youden’s Index: Sensitivity + Specificity − 1; 4 Indicators: Including ApoA1, ApoA2, LCA, and CEA. Significant values are in bold.

#### Analysis of model efficacy

**Figure 5:**
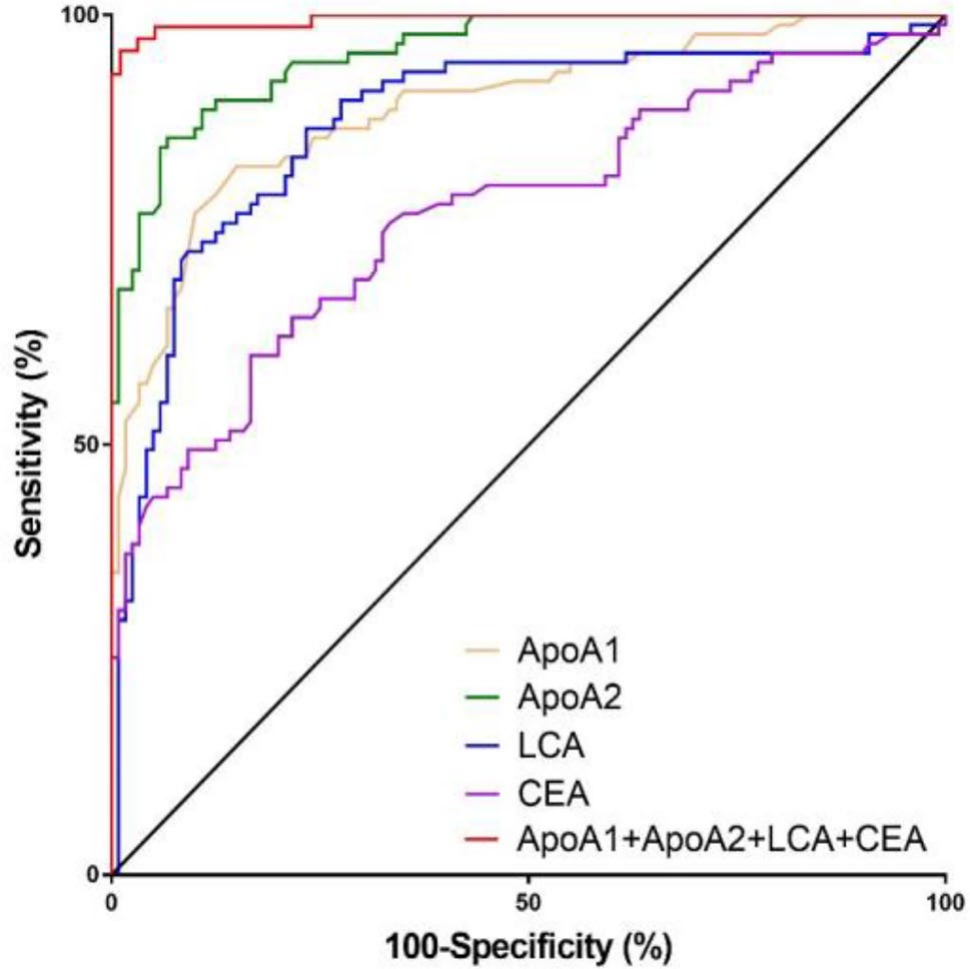
ROC curve of the model and the markers that make up the model in screening CRC.

The performance of Model Y was assessed using the ROC curve as a new variable. The AUC for diagnosing CRC was 0.995 (95% CI 0.969–0.999), as illustrated in Fig. 5. The model exhibited a sensitivity of 94.44%, a specificity of 97.92%, and an accuracy rate of 96.43%, as detailed in Table 4.

#### Internal validation of the model

**Figure 6:**
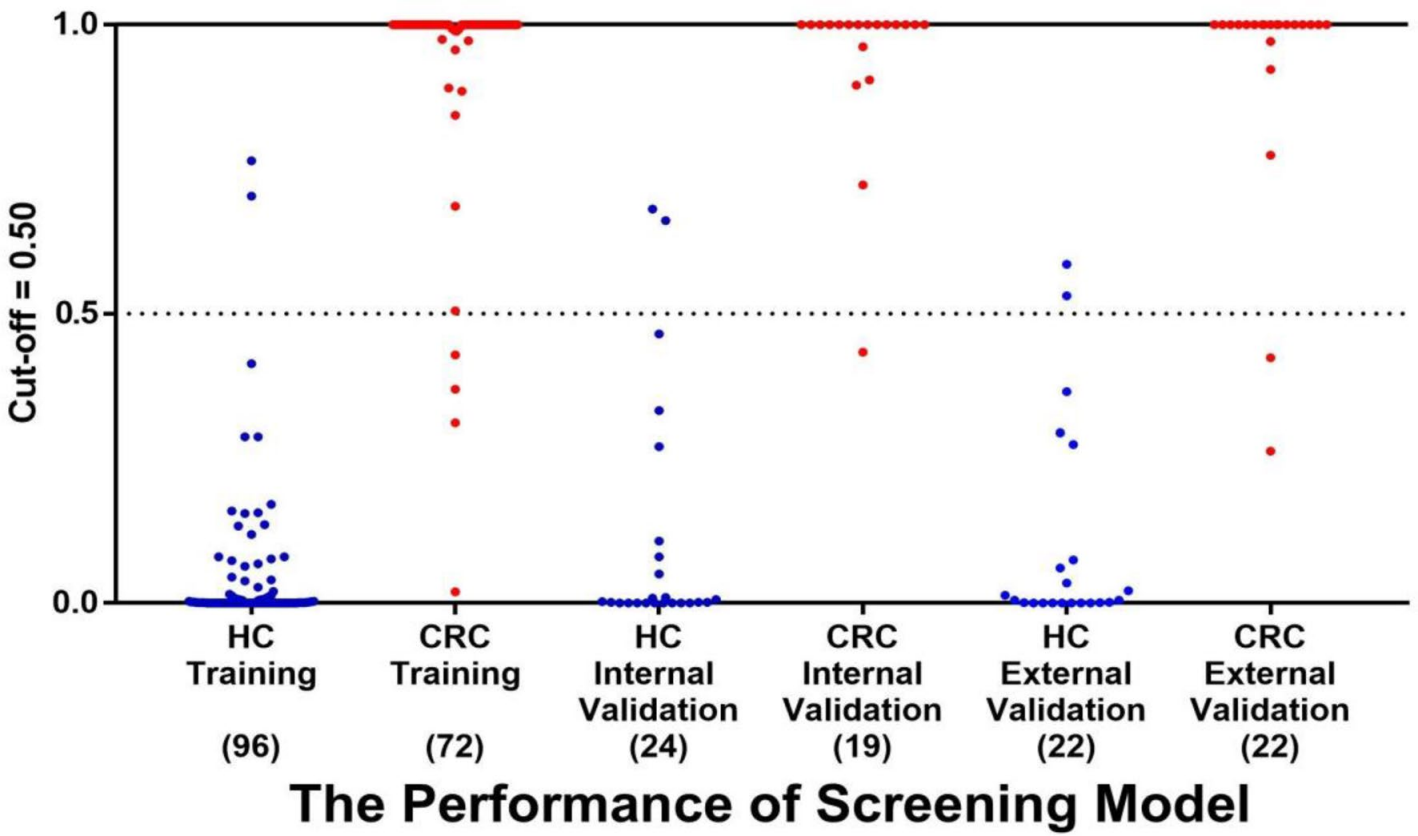
The performance of the model in screening CRC. *HC* Healthy control, *CRC* Colorectal cancer.

The other 20% of study participants’ samples (24 cases from the HC group and 19 cases from the CRC group) were utilized as internal validation set and assessed using Model Y. The results showed that 22 cases from the HC group and 18 cases from the CRC group were correctly classified, yielding a sensitivity of 94.74%, a specificity of 91.67%, and an overall accuracy rate of 93.02%. This accuracy rate was essentially consistent with that of the training set, as depicted in Fig. 6.

#### External validation of the model

For external validation of our findings, we recruited an additional 22 CRC patients and 22 healthy volunteers to form an independent validation set. Then evaluated Model Y's diagnostic accuracy on this external validation set. The results showed that 20 cases from CRC patients and 20 cases from healthy volunteers were correctly classified, achieving a sensitivity and specificity of 90.91% each. Figure 6 illustrated these results in detail.

## Discussion

Currently, the most prevalent CRC screening methods are fecal immunochemical testing (FIT) and colonoscopy. While FIT offers a non-invasive approach, it faces challenges of high false positive rates and low patient compliance during sample collection^13,14^. Colonoscopy, on the other hand, is not ideal for large-scale screening due to its invasive nature and potential complications. In CRC programmatic screening programs, colonoscopy is best reserved as step two of a two-stage screening cascade^7,8^. The strategy for the screening test in the first step of the screening cascade must consider feasibility, such as convenience and cost-effectiveness.

Blood testing emerges as a promising avenue for large-scale screening due to its minimally invasive nature. A promising strategy involves the development of multivariate classification models that integrate measurements of multiple biomarkers to calculate disease probability^15,16^. Such models can outperform single-analyte approaches by comprehensively capturing the intricate and multifaceted nature of cancer development, as well as the diverse metabolic, genetic, and structural alterations associated with cancer cells^17,18^. The application of multivariate models in cancer diagnosis is gaining traction, with numerous examples showcasing their effectiveness. FDA (U.S. Food and Drug Administration)-approved tests already exist, including a blood test based on multiple biomarkers for ovarian cancer detection and a multitarget stool DNA test for CRC screening^19,20^.

Researchers have delved into the potential of blood-based markers for CRC screening. For instance, Butvilovskaya et al.^21^ developed a CRC blood screening model employing tumor markers such as CEA, CA19-9, and CA125, achieving an AUC of 0.82. Similarly, Marín-Vicente et al.^22^ crafted a CRC screening model utilizing two serum protein biomarkers, ApoC3 and THBS1, analyzed through a decision tree algorithm, with an AUC of 0.83. However, the scope of indicators included in these models was somewhat limited, restricting significant advancements in diagnostic accuracy. Incorporating a variety of range of biomarkers into screening models emerges as a crucial strategy for the widespread, early detection of CRC. The inclusion of a diverse array of indicators—ranging from protein biomarkers to metabolic and transcriptomic biomarkers—enhances the model's diagnostic accuracy. Vironova et al.^23^ integrated 16 serum biomarkers into their model, resulting in exceptional diagnostic accuracy, with accuracy exceeding 95% and an AUC of over 0.98. Nevertheless, the model is overly complex for clinical application, necessitating the testing of an excessive number of biomarkers, which in turn increases the burden on patients. Thus, finding an optimal balance between the diversity of indicators and the model's diagnostic capability is a critical consideration. Additionally, ensuring the diagnostic reliability of blood-based CRC screening models presents a further challenge. Bhardwaj et al.^15^, in their analysis of 36 studies on the subject, found that most lacked external validation of the tests they proposed, highlighting an area in need of more focused attention.

In our study, we employed a comprehensive approach by integrating multiple indicators, including 9 lipids, 15 bile acids, and 6 gastrointestinal tumor markers, to simultaneously establish a screening model, which was not widely discussed in existing research. This model was refined to a core set of four high contribution indicators through stepwise logistic regression analysis. Compared to individual markers, our model demonstrates superior diagnostic accuracy with a sensitivity of 94.44%, a specificity of 97.92%, and an overall accuracy of 96.45% when the cut-off value is set at 0.5. It enables a more precise differentiation between healthy individuals and those with CRC. Importantly, the cut-off value can be adjusted according to specific clinical needs to prioritize sensitivity or specificity. For instance, a cut-off value of 0.3 increases the sensitivity to 98.61% while slightly reducing the specificity to 92.31%, as detailed in Supplementary Table 6. This adaptability allows for customized applications in different diagnostic scenarios. To ensure the model's robustness, we conducted external validation, achieving an accuracy of 90.91%. Another key advantage of the model is its flexibility. The four routine detection indicators included in the model—ApoA1, ApoA2, LCA, and CEA—can be easily incorporated into standard examinations to meet the needs of large-scale screening.

We recognize that different stages of colorectal cancer and other pathological conditions may influence the levels of circulating biomarkers, which was not involved in this study. To overcome this limitation, future research will include patients with various stages of CRC, those with gastrointestinal conditions such as inflammatory bowel disease, irritable bowel syndrome, hemorrhoids and colonic polyps, as well as those with other systemic diseases including dyslipidemia, liver, renal or heart diseases, inflammatory conditions or other cancers. A comprehensive comparative analysis of multi-omics biomarkers, including proteins, metabolites, and miRNAs, will be conducted. This enhances the practicality and applicability of the model. Moreover, we aim to achieve an optimal balance between the diversity of indicators and the diagnostic accuracy of the model. Our model will be refined by incorporating markers abnormally expressed in CRC serum, such as microRNA^24^, Midkine^25^, amino acid^26^ and other proteins such as albumin^27^. In addition, the sample size of our study is relatively small, and we intend to increase the sample size to further strengthen the validation of our model.

The ultimate validation of our model’s clinical utility will depend on conducting prospective randomized clinical trials. This step is crucial to ensure that our model is not just theoretically robust but also practically viable in a clinical setting.

### Supplementary Information

Supplementary Tables.

## Data Availability

The datasets analyzed during the current study are available from the corresponding author on reasonable request.
