# Supplementary material for: Exploration of the application potential of serum multi-biomarker model in colorectal cancer screening
Source: Sci Rep. 2024 May 2;14:10127. doi: 10.1038/s41598-024-60867-0 (PMC11066011; doi:10.1038/s41598-024-60867-0)
Supplement: Supplementary file 1 — Supplementary Tables. [file 41598_2024_60867_MOESM1_ESM.docx]

**Supplementary Materials**

**Supplementary Tab.1 Results for the indicators according to AJCC staging**

| Indicators | Stage I | Stage II | Stage III | Stage IV |
| --- | --- | --- | --- | --- |
| TC (mmol/L) | 4.58±1.18 | 4.72±0.70 | 4.45±0.87 | 4.33±0.56 |
| TG (mmol/L) | 1.11 (0.92~1.71) | 1.54(0.90~1.95) | 1.30 (0.92~1.94) | 1.40 (0.68~1.62) |
| HDL-C (mmol/L) | 1.26±0.34 | 1.25±0.30 | 1.17±0.37 | 1.19±0.31 |
| LDL-C (mmol/L) | 2.78±1.01 | 2.81±0.63 | 2.67±0.67 | 2.60±0.62 |
| ApoA1 (g/L) | 1.19±0.27 | 1.16±0.24 | 1.13±0.26 | 1.12±0.31 |
| ApoA2 (mg/dL) | 20.71±3.59 | 20.01±4.78 | 19.24±4.80 | 17.72±4.30 |
| ApoB (g/L) | 0.89±0.18 | 0.89±0.20 | 0.81±0.20 | 0.82±0.19 |
| ApoC2 (mg/dL) | 4.35 (2.99~5.51) | 4.21 (2.98~6.32) | 3.79 (2.94~5.22) | 3.45 (2.59~4.32) |
| ApoC3 (mg/dL) | 8.28 (6.91~9.44) | 9.23 (7.48~10.30) | 7.38 (6.71~9.56) | 7.10 (6.36~7.87) |
| CA (nmol/L) | 46.29 (17.08~112.50) | 49.32 (13.41~107.85) | 41.42 (22.93~230.36) | 15.17 (7.17~88.31) |
| GCA (nmol/L) | 104.45 (57.29~341.58) | 119.44 (37.90~341.61) | 160.94 (54.12~511.04) | 104.27 (24.73~144.45) |
| TCA (nmol/L) | 12.12 (6.09~38.39) | 10.29 (6.48~40.94) | 18.20 (5.20~39.54) | 8.80 (6.80~15.89) |
| CDCA (nmol/L) | 102.13 (36.15~365.73) | 268.65 (39.38~696.04) | 121.60 (22.41~914.53) | 38.69 (9.73~175.23) |
| GCDCA (nmol/L) | 670.75 (286.58~1060.65) | 770.20 (169.67~1497.02) | 666.28 (245.78~1515.60) | 427.73 (89.56~744.24) |
| TCDCA (nmol/L) | 63.33 (33.48~135.23) | 77.88 (19.54~182.05) | 75.04 (28.70~151.42) | 58.58 (17.81~179.18) |
| DCA (nmol/L) | 65.64 (17.09~272.56) | 84.65 (3.89~327.32) | 66.68 (8.66~277.45) | 51.79 (5.48~132.28) |
| GDCA (nmol/L) | 104.21 (6.22~328.43) | 47.39 (4.56~222.65) | 85.44 (14.92~240.86) | 56.31 (4.69~247.13) |
| TDCA (nmol/L) | 10.29 (1.13~29.26) | 3.00 (1.16~23.81) | 9.75 (1.13~27.07) | 9.76 (1.12~58.45) |
| LCA (nmol/L) | 7.86 (0.59~16.14) | 3.31 (0.01~9.57) | 5.76 (0.60~12.88) | 4.64 (0.79~8.42) |
| GLCA (nmol/L) | 0.46 (0.01~6.79) | 1.22 (0.01~4.73) | 1.33 (0.01~3.07) | 2.40 (0.01~15.97) |
| TLCA (nmol/L) | 0.45 (0.25~0.87) | 0.39 (0.24~1.11) | 0.34 (0.23~1.06) | 0.47 (0.09~3.95) |
| UDCA (nmol/L) | 13.10 (2.91~126.64) | 37.70 (5.17~131.48) | 7.62 (0.01~82.46) | 12.78 (0.13~26.88) |
| GUDCA (nmol/L) | 68.24 (17.78~161.82) | 53.62 (15.62~265.97) | 29.91 (10.12~182.96) | 60.68 (3.44~145.41) |
| TUDCA (nmol/L) | 1.71 (0.01~4.71) | 0.61 (0.01~7.67) | 0.01 (0.01~1.82) | 1.84 (0.01~5.86) |
| CA125 (U/mL) | 10.60(7.43~14.90) | 12.00 (8.39~15.45) | 10.11 (7.40~19.53) | 16.40 (15.40~27.40) |
| CA19-9 (U/mL) | 9.19 (5.69~12.80) | 9.69 (5.98~15.00) | 17.15 (6.26~31.83) | 15.40 (5.78~173.00) |
| CA242 (U/mL) | 3.58 (2.79~4.60) | 5.62 (3.33~7.63) | 8.29 (3.23~17.94) | 10.59 (2.83~200.00) |
| CA50 (U/mL) | 4.84 (3.56~6.69) | 6.15 (3.99~9.57) | 8.62 (4.18~16.51) | 6.64 (2.76~215.10) |
| CA72-4 (U/mL) | 1.52 (1.50~3.46) | 1.61 (1.50~2.62) | 1.91 (1.50~4.61) | 3.95 (1.63~9.83) |
| CEA (ng/mL) | 2.08 (1.73~3.02) | 3.83 (2.07~8.85) | 3.81 (1.78~9.17) | 5.80 (2.57~293.00) |

**Supplementary Tab.2 Results for the indicators according to tumor location**

| Indicators | Rectum | Colon |
| --- | --- | --- |
| TC (mmol/L) | 4.52±0.92 | 4.62±0.79 |
| TG (mmol/L) | 1.51 (0.92~1.91) | 1.12 (0.76~1.75) |
| HDL-C (mmol/L) | 1.19±0.32 | 1.28±0.37 |
| LDL-C (mmol/L) | 2.74±0.78 | 2.70±0.65 |
| ApoA1 (g/L) | 1.16±0.27 | 1.11±0.23 |
| ApoA2 (mg/dL) | 19.83±4.51 | 19.06±4.43 |
| ApoB (g/L) | 0.84±0.20 | 0.87±0.19 |
| ApoC2 (mg/dL) | 3.90 (3.05~5.27) | 3.59 (2.59~5.53) |
| ApoC3 (mg/dL) | 7.66 (6.78~9.43) | 8.28 (6.90~10.62) |
| CA (nmol/L) | 38.67 (16.39~117.22) | 56.38 (9.69~266.79) |
| GCA (nmol/L) | 105.51 (43.16~429.48) | 144.45 (42.08~310.36) |
| TCA (nmol/L) | 12.22 (5.68~37.84) | 15.89 (6.72~31.82) |
| CDCA (nmol/L) | 113.37 (30.10~419.67) | 175.23 (27.55~803.32) |
| GCDCA (nmol/L) | 600.80 (270.61~1324.52) | 731.24 (136.02~1401.84) |
| TCDCA (nmol/L) | 63.25 (28.32~154.46) | 78.63 (22.89~179.18) |
| DCA (nmol/L) | 68.96 (10.21~228.67) | 64.68 (1.74~291.98) |
| GDCA (nmol/L) | 74.97 (15.45~240.86) | 60.41 (0.94~307.67) |
| TDCA (nmol/L) | 8.45 (1.36~25.87) | 4.07 (0.69~28.66) |
| LCA (nmol/L) | 5.73 (0.86~13.01) | 4.69 (0.01~14.44) |
| GLCA (nmol/L) | 1.43 (0.01~3.91) | 0.01 (0.01~6.56) |
| TLCA (nmol/L) | 0.43 (0.22~1.04) | 0.37 (0.30~1.34) |
| UDCA (nmol/L) | 12.94 (0.25~88.21) | 23.53 (0.13~112.19) |
| GUDCA (nmol/L) | 47.51 (15.38~190.66) | 53.62 (9.31~222.27) |
| TUDCA (nmol/L) | 0.15 (0.01~4.55) | 0.01 (0.01~6.07) |
| CA125 (U/mL) | 10.70 (7.57~16.08) | 14.30 (8.67~17.10) |
| CA19-9 (U/mL) | 9.94 (6.17~17.18) | 13.10 (5.68~20.40) |
| CA242 (U/mL) | 4.87 (2.85~11.52) | 7.76 (3.60~16.18) |
| CA50 (U/mL) | 5.75 (3.92~11.45) | 8.56 (4.86~16.62) |
| CA72-4 (U/mL) | 1.66 (1.50~3.94) | 1.78 (1.50~4.62) |
| CEA (ng/mL) | 2.88 (1.81~6.95) | 4.15 (2.23~9.27) |

**Supplementary Tab.3** **Comparison of biomarker sensitivities at different specificities**

| Indicators | Biomarker Sensitivities at Different Specificities (%) | | | | |
| --- | --- | --- | --- | --- | --- |
|  | 90% | 80% | 70% | 60% | 50% |
| TC | 31.87 | 53.85 | 59.34 | 69.23 | 71.43 |
| TG | 7.69 | 24.18 | 34.07 | 42.86 | 52.75 |
| HDL-C | 24.18 | 47.25 | 58.24 | 65.93 | 70.33 |
| LDL-C | 17.58 | 36.26 | 53.85 | 61.54 | 73.63 |
| APOA1 | 76.92 | 82.42 | 86.81 | 91.21 | 92.31 |
| APOA2 | 86.81 | 92.31 | 95.60 | 97.80 | 100.00 |
| APOB | 17.58 | 30.77 | 51.65 | 58.24 | 70.33 |
| APOC2 | 18.68 | 29.67 | 43.96 | 53.85 | 59.34 |
| APOC3 | 47.25 | 58.24 | 61.54 | 64.84 | 78.02 |
| CA | 29.67 | 46.15 | 54.95 | 64.84 | 67.03 |
| CDCA | 47.25 | 54.95 | 62.64 | 73.63 | 80.22 |
| DCA | 56.04 | 71.43 | 76.92 | 80.22 | 86.81 |
| GDCA | 43.96 | 56.04 | 58.24 | 67.03 | 70.33 |
| TDCA | 52.75 | 63.74 | 67.03 | 75.82 | 79.12 |
| LCA | 71.43 | 78.02 | 90.11 | 92.31 | 92.31 |
| GLCA | 53.85 | 68.13 | 75.82 | 75.82 | 82.42 |
| TLCA | 21.98 | 54.95 | 67.03 | 71.43 | 76.92 |
| UDCA | 47.25 | 62.64 | 71.43 | 74.73 | 78.02 |
| GUDCA | 40.66 | 58.24 | 59.34 | 64.84 | 69.23 |
| TUDCA | 53.85 | 60.44 | 67.03 | 70.33 | 73.63 |
| CA125 | 14.29 | 26.37 | 37.36 | 48.35 | 56.04 |
| CA19-9 | 26.37 | 42.86 | 51.65 | 63.74 | 64.84 |
| CA242 | 26.37 | 32.97 | 40.66 | 47.25 | 48.35 |
| CA50 | 21.98 | 29.67 | 38.46 | 48.35 | 57.14 |
| CA19-9 | 26.37 | 42.86 | 51.65 | 63.74 | 64.84 |
| CEA | 49.45 | 64.84 | 71.43 | 80.22 | 83.52 |

**Supplementary Tab.4 Clinical characteristics of the training set and internal validation set**

|  | **Study participants** | | | |
| --- | --- | --- | --- | --- |
|  | **Training set** | | **Internal validation set** | |
| Group | HC | CRC | HC | CRC |
| Total (%) | 96(100.00) | 72(100.00) | 24(100.00) | 19(100.00) |
| Age distribution | | | | |
| <50 (%) | 7(7.3) | 5(6.9) | 2(9.4) | 1(5.3) |
| 50-59 (%) | 28(29.2) | 12(16.7) | 10(41.7) | 4(21.0) |
| 60-69 (%) | 35(36.4) | 29(40.3) | 7(29.1) | 9(47.4) |
| >70 (%) | 26(27.1) | 26(36.1) | 5(20.8) | 5(26.3) |
| Median(years) | 66.00 | 67.00 | 59.00 | 63.00 |
| Mean(years) | 63.81 | 65.49 | 61.58 | 63.37 |
| SD | 8.91 | 9.90 | 9.25 | 9.79 |
| Gender distribution | | | | |
| Male (%) | 62(64.6) | 44(61.1) | 15(62.5) | 14(73.7) |
| Female (%) | 34(35.4) | 28(38.9) | 9(37.5) | 5(26.3) |
| Stage distribution^#^ | | | | |
| I (%) | — | 18(25.0) | — | 5(26.3) |
| II (%) | — | 20(27.8) | — | 5(26.3) |
| III (%) | — | 24(33.3) | — | 8(42.1) |
| IV (%) | — | 10(13.9) | — | 1(5.3) |
| Tumor location distribution | | | | |
| Rectum (%) | — | 31(43.1) | — | 8(42.1) |
| Colon (%) | — | 41(56.9) | — | 11(57.9) |

HC: Healthy control; CRC: Colorectal cancer. # According to American Joint Committee on Cancer (AJCC) Cancer Staging Manual Eighth Edition

**Supplementary Tab.5 Comparison of two models after removing the indicator with the lowest contribution**

| Model  (Step) | Indicators in model | Removing indicator | Contribution for Indicator | *P* vale  (vs. next model) |
| --- | --- | --- | --- | --- |
| 1 | ApoA1+ApoA2+ApoC3+CDCA+DCA+TDCA +LCA+GLCA +UDCA+TUDCA+CEA | DCA | 0.006 | 0.938 |
| 2 | ApoA1+ApoA2+ApoC3+CDCA+TDCA +LCA+GLCA +UDCA+TUDCA+CEA | TUDCA | 0.935 | 0.333 |
| 3 | ApoA1+ApoA2+ApoC3+CDCA+TDCA +LCA +GLCA +UDCA+CEA | TDCA | 0.886 | 0.347 |
| 4 | ApoA1+ApoA2+ApoC3+CDCA +LCA+GLCA +UDCA+CEA | UDCA | 1.504 | 0.220 |
| 5 | ApoA1+ApoA2+ApoC3+CDCA +LCA+GLCA +CEA | CDCA | 0.716 | 0.397 |
| 6 | ApoA1+ApoA2+ApoC3 +LCA+GLCA +CEA | APOC3 | 1.379 | 0.240 |
| 7 | ApoA1+ApoA2+LCA+GLCA +CEA | GLCA | 1.699 | 0.192 |
| **8** | **ApoA1+ApoA2+LCA+CEA** | **ApoA1** | **5.100** | **0.024** |
| 9 | ApoA2+LCA+CEA | LCA | 23.591 | <0.001 |
| 10 | ApoA2+CEA | CEA | 25.796 | <0.001 |
| 11 | ApoA2 | — | — | — |

Removing indicator: The indicator would be removed in next step. Contribution for Indicator: The model’s change in -Log_2_ Likelihood after removing this indicator; *P* vale: According to Likelihood ratio test.

**Supplementary Tab.6** **Sensitivity and specificity corresponding to different cut-off value in each set**

| Cut-off | Training Set | | Internal Validation Set | | External Validation Set | |
| --- | --- | --- | --- | --- | --- | --- |
|  | Sensitivity (%) | Specificity (%) | Sensitivity (%) | Specificity (%) | Sensitivity (%) | Specificity (%) |
| 0.8 | 91.67 | 100.00 | 89.47 | 100.00 | 86.36 | 100.00 |
| 0.7 | 91.67 | 97.92 | 94.74 | 100.00 | 90.91 | 100.00 |
| 0.6 | 93.06 | 97.92 | 94.74 | 91.67 | 90.91 | 100.00 |
| 0.5 | 94.44 | 97.92 | 94.74 | 91.67 | 90.91 | 90.91 |
| 0.4 | 95.83 | 96.88 | 100.00 | 87.50 | 95.45 | 90.91 |
| 0.3 | 98.61 | 96.88 | 100.00 | 83.33 | 95.45 | 86.36 |
| 0.2 | 98.61 | 94.79 | 100.00 | 79.17 | 100.00 | 77.27 |

Cut-off: Probability of occurrence;
